# Predictive Modelling of H_2_S Removal from Biogas Generated from Palm Oil Mill Effluent (POME) Using a Biological Scrubber in an Industrial Biogas Plant: Integration of Artificial Neural Network (ANN) and Process Simulation^§^

**DOI:** 10.17113/ftb.63.02.25.8792

**Published:** 2025-06

**Authors:** Joanna Lisa Clifford, Yi Jing Chan, Mohd Amran Bin Mohd Yusof, Timm Joyce Tiong, Siew Shee Lim, Chai Siah Lee, Woei-Yenn Tong

**Affiliations:** 1 Department of Chemical and Environmental Engineering, University of Nottingham Malaysia, Broga Road, 43500, Semenyih, Selangor Darul Ehsan, Malaysia; 2 Advanced Materials Research Group, Faculty of Engineering, University of Nottingham, NG7 2RD, UK; 3 Universiti Kuala Lumpur, Institute of Medical Science Technology, A1-1, Jalan TKS 1, Taman Kajang Sentral, 43000 Kajang, Selangor, Malaysia

**Keywords:** palm oil mill effluent, biogas, simulation, artificial neural network, bioscrubber

## Abstract

**Research background:**

Biogas production from palm oil mill effluent (POME) is inherently unstable due to variations in feedstock composition and operating conditions. These fluctuations can lead to inconsistent biogas quality, variable methane content and fluctuating hydrogen sulphide (H_2_S) concentration. This poses significant challenges for bioscrubbers in removing H_2_S to meet quality standards for gas engines used for electricity generation. This research aims to address these challenges by integrating simulation models with a computer programme and artificial neural network (ANN) to predict the performance of a bioscrubber at a POME treatment plant in Johor, Malaysia.

**Experimental approach:**

First, the process flowsheet model was simulated using a computer programme. The H_2_S removal was then predicted using a machine learning algorithm, specifically ANN, based on two years of historical data obtained from the biogas plant. A detailed techno-economic analysis was also carried out to determine the economic feasibility of the process.

**Results and conclusions:**

The simulation results showed a biogas yield of 26.12 Nm^3^ per m^3^ POME, which is in line with industry data with less than 1 % deviation. The ANN model achieved a high coefficient of determination (R^2^) of 0.9 and a low mean squared error (MSE), with the bioscrubber reaching an H_2_S removal efficiency of approx. 96 %. The techno-economic analysis showed that the process is feasible with a net present value (NPV) of $131 000 and a payback period of 7 years.

**Novelty and scientific contribution:**

The integration of ANN and the computer programme provides a robust framework for predicting bioscrubber performance and ensuring stable bioscrubber operation due to their complementary strengths. ANN accurately predicts H_2_S removal based on daily recorded data, while the computer programme estimates parameters that are not monitored daily, such as chemical oxygen demand (COD), biological oxygen demand (BOD) and total suspended solids (TSS). This research provides valuable insights into sustainable biogas production practices and offers opportunities to improve energy efficiency and environmental sustainability in the palm oil industry.

## INTRODUCTION

Palm oil is an edible vegetable oil popular in tropical areas, which is obtained from the mesocarp of the fruit of oil palm trees (*1*). Malaysia has become one of the leading producers and exporters of the palm oil in the world. The palm oil industry has been a stable contributor to the growth of Malaysian economy. According to the Malaysian Palm Oil Board, the total land usage for palm oil cultivation in 2022 was estimated to approx. 5.67 million hectares. In 2022, the production of crude palm oil (CPO) in Malaysia was 18.45 million tonnes and is projected to increase by 3 % in 2023 (*2*). However, the potential environmental impacts due to high production rates are becoming more significant. With the number of palm oil mills increasing each year, the increase in by-products such as fresh fruit bunch waste and effluent discharge is inevitable (*3*).

Palm oil mill effluent (POME) is the wastewater produced during palm oil milling activities, mainly during oil extraction, washing and cleaning processes. Due to its highly polluting properties, POME needs to be properly treated before it is discharged into the environment (*4*). POME is a viscous, brown liquid with a pH of 4 to 5. It is 100 times more polluting than municipal sewage, which has a high chemical oxygen demand (COD) and biological oxygen demand (BOD) (*3*). POME is one of the biggest problems in the production industry due to the large amounts of waste produced annually and its disposal issues (*5*). Therefore, POME treatment deserves more attention and emphasis to promote sustainability and circular economy in the industry.

There are several common effluent treatment systems used in the palm oil industry, such as ponding systems, covered lagoon systems, closed anaerobic digesters and land application systems (*6*). Among these techniques, biological treatment is the most popular method for treating POME in many palm oil mills (*7*). Most palm oil mills have implemented anaerobic digestion (AD) as the primary treatment for POME. The AD process usually operates at a temperature of 30-65 °C (*8*). During anaerobic digestion, the biodegradation of POME releases a combustible methane-rich gas that is captured and converted to renewable energy (*9*).

Biogas is a renewable energy source obtained from the anaerobic digestion of POME in the absence of oxygen. Anaerobic digestion is a multi-step biochemical process of converting organic material into biogas. It is a natural fermentation process in which bacteria break down the organic matter into its components until all that is left are gases and a residue called digestate (*10*). Biogas can be used to produce heat and electricity with minimum impact on the climate and is a sustainable solution for organic waste management. It is a promising alternative to fossil fuels (*11*). The chemical composition of biogas is influenced by the properties of raw materials and the conditions under which AD is carried out. The sources for biogas production include a wide range of inputs such as wastewater, sewage sludge, animal or agricultural waste and landfill materials (*12*).

Biogas consists mainly of methane and carbon dioxide as well as trace amounts of nitrogen, oxygen, hydrogen sulphide and water. The biogas produced in the palm oil industry is mostly used to generate electricity. The main disadvantage of biogas is the presence of H_2_S (*13*). Maintaining low H_2_S concentrations is essential for environmental, health and safety reasons. The H_2_S concentration in unprocessed biogas ranges from 50 to 10 000 mg/L, depending on the properties of POME. H_2_S causes the corrosion of various steels, including stainless steel and other copper and nickel alloys, thus high concentrations can damage the equipment and increase the costs. It is also particularly corrosive to gas engines and shortens the life of the engine. This, in turn, increases service and maintenance costs and reduces the conversion of biogas to electricity. Therefore, the recommended H_2_S concentration in biogas is in the range of 200 to 500 mg/L, depending on engine specifications (*14*). Furthermore, high concentrations of H_2_S in the atmosphere can pollute the environment and pose serious health risks, as H_2_S is toxic and in high concentrations can cause respiratory and neurological problems in humans. In addition, unregulated H_2_S emissions contribute to a pungent odour that affects surrounding communities and the environment, potentially violating air quality standards.

Therefore, the efficient removal of H_2_S from biogas is crucial for the proper functioning of the gas engine and compliance with regulatory standards. Bioscrubbers are used to remove H_2_S from biogas in most palm oil mills. Microbial activity in bioscrubbers is observed to degrade contaminants in the biogas such as H_2_S before it enters the gas engine (*15*). Biological approaches are favoured due to their lower operating costs than of physical and chemical methods. Moreover, they do not generate secondary streams. The minimal use of chemicals also makes the biological approach more cost-effective and environmentally friendly than physicochemical and chemobiological methods.

Despite the potential of bioscrubbers, the lack of comprehensive simulation data is a major challenge for biogas plants, such as the plant in Johor, Malaysia, which serves as a case study in this research. Biogas production from POME is inherently unstable due to variations in feedstock composition, operating conditions and other factors (*16*). These fluctuations can lead to inconsistent biogas quality, variable methane content and fluctuating concentrations of H_2_S, which subsequently affect the efficiency of the bioscrubber. The plant in Johor has reported that due to these fluctuations and the inherent variability of the biological processes, it is difficult to achieve consistent H_2_S removal with their current bioscrubber. Factors such as temperature, pH, microbial community dynamics and the concentration of H_2_S and other compounds can change unpredictably, leading to inconsistent performance. The complexity of microbial interactions and their sensitivity to environmental changes make it challenging to maintain optimal H_2_S removal. Therefore, advanced solutions are required to manage and mitigate these fluctuations.

Simulation plays a crucial role in overcoming this instability by providing means to predict and analyse the behaviour of bioscrubbers under different operating conditions with minimum operating costs. By simulating bioscrubber performance, researchers and engineers can assess how variations in parameters such as inlet gas composition, flow rate and temperature affect the efficiency of H_2_S removal. In this way, optimal operating conditions can be identified that maximise H_2_S removal efficiency while minimising energy consumption and operational costs. Furthermore, simulation allows the evaluation of bioscrubber performance over time and under different conditions, providing insights into its long-term effectiveness and reliability. This is particularly important in the context of biogas plants, where consistent and reliable performance is essential for meeting regulatory requirements and ensuring sustainable operation of the facility.

Yap and Hasanah (*17*) conducted a simulation study on a water scrubber using ChemCAD simulation and showed the feasibility of purifying POME-based biogas to produce high-purity methane. However, further optimisation and economic analysis of the process was not carried out. This highlights the need for comprehensive studies that integrate both technical and economic aspects to assess the feasibility and viability of biogas purification processes. SuperPro Designer, v. 9.0 (*18*) is a versatile tool widely used for the simulation of complex industrial processes, including those related to anaerobic digestion and biogas treatment systems (*19*). It enables detailed modelling of process streams and provides essential data such as COD values, while facilitating energy and mass balance calculations and incorporating critical reactions in anaerobic digestion. For POME treatment, this software was used to analyse biogas production, treatment and wastewater stabilisation. Chong *et al.* (*20*) used it to evaluate the performance of an integrated anaerobic-aerobic bioreactor (IAAB) and optimise parameters for improved biogas yield and COD removal. Similarly, Kan *et al.* (*21*) demonstrated its accuracy in predicting treatment outcomes with less than 3 % error between simulated and experimental data. Despite its versatility, previous studies have not fully explored the role of bioscrubber systems and H_2_S removal in meeting the requirements of gas engine. This highlights the potential of using SuperPro Designer, v. 9.0 (*18*) for the simulation of industrial-scale POME treatment processes, with a focus on the integration of bioscrubbers.

Machine learning (ML) has advanced over the years and it can be used as a prediction tool in the studies of microbial ecology and system biology (*22*). ML tools have been used in a few studies to analyse and predict the performance of anaerobic digestion (*23*, *24*). Furthermore, the performance of biogas purification and H_2_S removal has been reported to be affected by the inlet temperature, operating pressure and biogas flow rate. Thus, to better understand the behaviour of biogas purification, ML tools can be used to predict the performance of H_2_S removal from biogas. The artificial neural network (ANN) approach is reported to be among the most widely used ML approaches (*25*). However, the application of ANN to bioscrubbers poses a challenge, including the need for high-quality data due to the fluctuating conditions under which bioscrubbers operate, such as varying inlet gas composition, temperature and pressure. This variability can lead to inaccurate predictions if not properly accounted for. Additionally, non-linear interactions between bioscrubber parameters, such as pH and microbial activity, require advanced ANN architectures and precise model tuning. Overcoming these challenges requires increased data collection, model refinement and real-time operational feedback to improve the accuracy and reliability of ANN predictions. Most importantly, there is little research on ANN-based prediction models for H_2_S removal in bioscrubbers operating in real biogas plants.

Therefore, this research aims to fill this research gap by integrating simulation models using SuperPro, v. 9.0 (*18*) and ANN to predict the performance of a bioscrubber based on two years of data recorded at a POME treatment plant in Johor. The simulation results were compared with industry data to confirm the accuracy of the model. In addition, a techno-economic analysis was conducted to evaluate the feasibility and economic viability of the treatment plant, which included both the POME treatment and the biogas purification process. This study offers a pathway towards more sustainable biogas purification practices, with potential benefits for energy efficiency and environmental performance in the palm oil industry. In doing so, it supports key global priorities, particularly SDG 7 (Affordable and Clean Energy), SDG 12 (Responsible Consumption and Production), SDG 13 (Climate Action), and SDG 9 (Industry, Innovation and Infrastructure) (*26*).

## MATERIALS AND METHODS

**Fig. S1:**
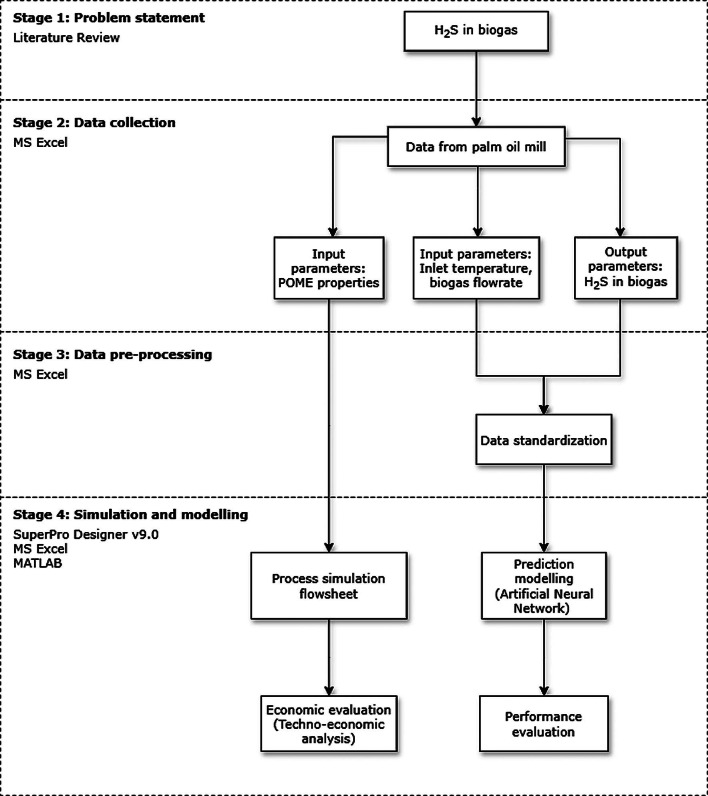
Framework for simulation and prediction modelling using machine learning and economic analysis. POME=palm oil mill effluent

This study investigated the removal of H_2_S from raw biogas using a bioscrubber in a palm oil mill effluent (POME) treatment plant. A simulation of the POME treatment process and biogas purification in an existing plant in Johor, Malaysia, was conducted to evaluate the feasibility of a bioscrubber. Furthermore, an optimisation approach using artificial neural networks (ANN) was applied to analyse the biogas purification process. The research framework shown in Fig. S1 was developed based on the actual plant data.

### Data collection

After identifying the problem and setting the study objectives, the next crucial step is data collection. Most data used in this research were obtained by collaboration with a palm oil mill in Johor, Malaysia. Input parameters, such as POME properties including chemical oxygen demand (COD), total solids (TS) and total volatile solids (TVS), were collected from the mill every month. Additionally, data on temperature, biogas flow rate, H_2_S concentrations and biogas composition were collected from their supervisory control and data acquisition (SCADA) system. The data for the machine learning model were collected over a period of two years, which provides detailed operational data for accurate model development and validation.

### Data preprocessing

A total of 90 industrial datasets were collected from the palm oil mill in Johor. To generate an effective model for performance analysis, it is essential to preprocess the data to obtain small variations in the output data. Theoretically, the output data usually have a wide range of data. However, if the output range is too large, the ANN model may appear less stable and reliable (*27*). The preprocessing of the data was carried out by a normalisation technique where the data were scaled to fit within a range of 0-1 using the following equation (*28*):


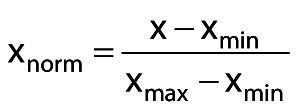
 /1/

where x is the variable, x_max_ is the maximum value and x_min_ is the minimum value.

Overfitting is a common problem that occurs when training neural networks. To solve this problem, the data were divided into three segments: training, testing and validation sets. With this approach, the validation error is constantly monitored and if there is any indication of an increase in it, the training is stopped (*28*). It was configured so that 70 % of the datasets were used to train the model. Meanwhile, 15 % was set for validation and testing, which confirms the accuracy of the machine learning model. The software used to run this ANN model was MATLAB R2022a (*29*). The neural network fitting in the software was the application of the programming language used to generate the ANN model.

### Process simulation

**Fig. S2:**
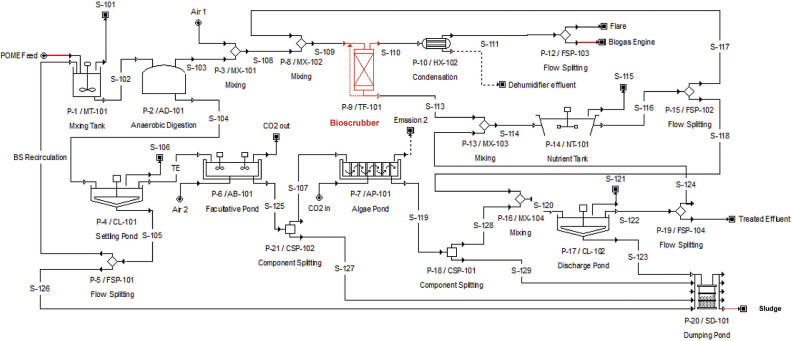
Simulated process flow sheet for biogas production from palm oil mill effluent (POME)

The simulation of the POME treatment and biogas purification processes was carried out using SuperPro Designer v. 9.0 (*18*). The input parameters and compositions for the simulation were obtained from the palm oil mill located in Johor, Malaysia. The composition of POME in the feed stream is shown in Table 1. The feed flow rate was obtained from the data collected from the plant. The flow rate of POME from the cooling pond that enters the mixing tank was recorded weekly. The process flow sheet and equipment selection were then simulated using the information provided by the plant. The sizing calculations performed on SuperPro Designer v. 9.0 (*18*) were completed after mass balance calculations had zero errors. The simulated process flow sheet is shown in Fig. S2.

**Table 1:** Palm oil mill effluent (POME) mass flow rate and feed composition used in the SuperPro (*18*) simulation

Component	Mass flow rate/(kg/h)	*w*(component)/%
Biomass	285.376	1.9865
Carbohydrate	285.878	1.9900
Fibre	7.743	0.0539
Nitrate	13.547	0.0943
Oil and grease	61.040	0.4249
Phosphate	1.839	0.0128
Protein	61.040	0.4249
Sulfate	1.839	0.0128
Water	13647.457	95.0000

As shown in Fig. S2, the POME feed enters a mixing tank (P-1) at a flow rate of 14 366 kg/h after leaving a cooling pond. The operating conditions of the mixing tank were set to a temperature of 57.3 °C and a pH=6.89. The mixture is then anaerobically digested in a covered anaerobic lagoon (P-2). The purpose of a covered digester is to contain the produced CH_4_ and other gases. The loss of these components could result in a significant loss of profit due to lower purity and emission of greenhouse gases. The biogas produced must then be further purified to remove undesired components such as H_2_S. The biogas is purified by removing H_2_S in the bioscrubber (P-9).

In this biogas plant, a low-pressure biological scrubber (LPBS) is used for H_2_S removal. The H_2_S is decomposed by oxidation, producing sulphur and water (*13*).

An LPBS usually consists of one or more gas-liquid contact columns connected to a recirculation tank consisting of water with nutrients. Each column is partially filled with packing media that are available in a range of shapes (*30*). The packing media are usually made of non-corrosive materials including glass, metal, ceramic or plastic, on which the microorganisms are immobilised. The shape of the packing media plays an important role in reducing the gas pressure drop as they provide a small contact area per unit volume and increase the void fraction for gas passage (*30*). Moreover, the packing media allow the microorganisms to form a biofilm (*31*). The biogas stream is channelled through the media, while the nutrient water is sprayed over them. The nutrient water is sourced from the settling pond after anaerobic digestion and contains only minimal solids (less than 1 %). Contaminants such as H_2_S are absorbed from the raw biogas into the liquid, where microorganisms grow on the media and biological oxidation occurs. Normally, bacteria of the genus *Thiobacillus* are used to remove H_2_S from biogas.

Next, the moisture content of this product stream is reduced using a chiller (P-10) (*32*). In the meantime, the digestate from the anaerobic digester enters a settling pond (P-4) to stabilise wastewater sludge before the bottom sludge is recirculated to the original mixing tank. The treated effluent is then aerobically treated in a facultative pond (P-6) and then in an algal pond (P-7) for further breakdown of biodegradable materials. The pond effluent then enters a discharge pond (P-17), where wastewater is further separated from suspended particles by sedimentation. The sludge is then passed to a dumping pond and sent to a composting plant (*33*).

### Neural network fitting

Machine learning (ML) is a discipline of artificial intelligence (AI) that trains machines or software to identify patterns and make predictions based on historical data with minimal human supervision. Over the years, advances in ML in the industry have shown great potential as a prediction tool in many studies. In the POME industry, it can be observed that the integration of ML is becoming more prevalent in quality estimation in recent years (*28*).

ANN consists of interconnected nodes or neurons linked by weights. Each node in parallel layers receives data from previous nodes, processes it through a nonlinear function and transmits the result to the subsequent nodes. The modelling process of an ANN involves several steps. The data in this study were recorded weekly and compiled every month from July 2021 to June 2023. A total of 90 datasets were used to develop the machine learning algorithm to obtain the prediction model. With the MATLAB (*29*) settings for neural network fitting of 70 % for validation and 15 % each for training and testing, the training datasets were used to train the ANN model for future predictions.

Hyperparameters define the structure and learning process of a neural network and play a crucial role in optimising the performance of an ANN model. Unlike model parameters, which are learned during training, hyperparameters such as the learning rate, the number of hidden layers and the number of neurons must be determined before the training process. In this study, random search was used to tune the hyperparameters, as it is more efficient and practical in determining optimal hyperparameter combinations than grid search. This approach helps to prevent overfitting or underfitting and ensures that the model generalises well to unseen data, leading to improved prediction accuracy.

Parameters that affect the performance of the bioscrubber, such as the type of packing medium, bacterial species, empty bed residence time and the recirculation ratio of liquid media (*34*), play a crucial role. Additionally, other factors such as pressure, biogas flow rate, operating pH and the shape of the packing media can influence the efficiency of the bioscrubber.

The inlet temperature and biogas flow rate were chosen as input data for the model because these parameters are routinely monitored and recorded daily by operators in biogas plants. In particular, the inlet temperature can affect the microbial activity in the bioscrubber, while the biogas flow rate affects the contact time between the biogas and the liquid medium, which affects the efficiency of H_2_S removal (*35*). The outlet H_2_S concentration was chosen as the output data and integrated into the output layer of the model. This is because it serves as a direct measure of the performance of the bioscrubber and reflects the effectiveness of H_2_S removal in the biogas stream. This concentration is a critical parameter to comply with environmental regulations and is essential for ensuring that the biogas meets quality standards for downstream applications.

### Economic evaluation

Techno-economic analysis (TEA) has become a crucial tool for assessing the economic potential of industrial processes, especially in the context of Industrial Revolution (IR) 4.0. As industry has evolved, TEA has increasingly integrated data-driven technologies such as artificial intelligence (AI) and blockchain to optimise processes and economic factors (*36*). In this study, process simulation is combined with TEA to evaluate the economic feasibility of the biogas plant using the bioscrubber. The analysis includes steps such as process design, equipment sizing, cost estimation and cash flow analysis. Data on plant operation, design, transport and market behaviour were collected from the biogas plant. Market behaviour refers to the trends in pricing and demand for biogas and its by-products, including the feed-in tariff (FiT) policies in Malaysia, which affect the economic viability of the project. After successful simulation and sizing of the equipment, economic calculations were conducted using updated prices from 2022. The economic evaluation function of the SuperPro software, v. 9.0 (*18*) facilitated the preparation of a comprehensive economic evaluation report.

### Statistical analysis

In this study, statistical analysis of mean squared error (MSE) was used as an indicator of the fitness of the model. MSE is determined during the neural network programming using the following equation:


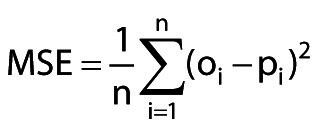
 /2/

where o is the observed value for the i-th data point (from experimental or real data) and p is the predicted value generated by the model for the i-th data point.

While the root mean square error (RMSE) is evaluated in the same unit, making it a more interpretable measure, the MSE is more effective in evaluating the distribution of the data and the overall measure of accuracy. This is because outliers have a greater impact as all the differences are squared and all errors are positive. This minimises the effect of negative and positive differences cancelling each other out.

The coefficient of determination (R^2^) is calculated in ANN modelling using the following equation:


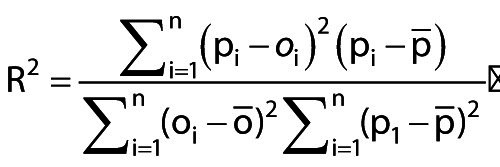
 /3/

The fitting of the model improves as R approaches 1.

## RESULTS AND DISCUSSION

### Simulation model

The simulated results including the composition of biogas from anaerobic digestion and its purified form are shown in Table 2. The simulation results show the final H_2_S concentrations in the biogas product to be 43 mg/L and the H_2_S removal efficiency by the bioscrubber was calculated to be approx. 95.5 %. This indicates that the operational conditions of the current palm oil mill are satisfactory, as the concentration of H_2_S in the biogas remains well below the recommended threshold of 500 mg/L. Overall, the difference of most parameters in Table 2 is less than 10 %, indicating that the simulation model accurately predicts the performance of the bioscrubber system for these parameters.

**Table 2:** Composition of biogas from anaerobic digestion (AD) and purified biogas

Parameter	Compound	Industry data	Simulated data	Relative difference/%
Raw biogas from AD	*φ*(CH_4_)/%	(60.5±1.0)	62	2.5
	*γ*(H_2_S)/(mg/L)	(943±264)	957	1.5
	*φ*(CO_2_)%	(34.8±1.1)	33	5.2
	*φ*(O_2_)/%	(0.6±0.1)	0.5	7.3
Purified biogas to gas engine	*γ*(H_2_S)/(mg/L)	(37±38)	43	16.2
Yield	*Y*(CH_4_)/(Nm^3^ biogas per m^3^ POME)	(25.7±6.6)	26.12	1.7
Scrubber efficiency (H_2_S removal)	*φ*(H_2_S)/%	(96.1±3.9)	95.5	0.6

Nm^3^=normal cubic meter (at *t*=0 °C and *p*=101.3 kPa), POME=palm oil mill effluent

### Results of artificial neural network model

**Fig. 1:**
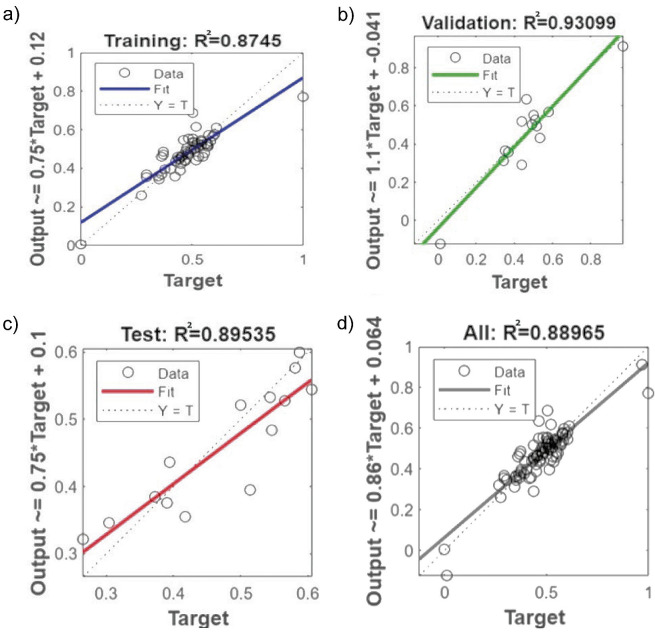
Regression analysis of the ANN model during: a) training, b) validation, c) testing and d) overall phase

The artificial neural network (ANN) adopts a multi-layer feed-forward neural network that uses the Levenberg-Marquardt training algorithm. The concept of feed-forward is to take measurements and corrective actions before the process is disturbed. The settings and properties of the prediction model are shown in Table 3. The four graphs in Fig. 1 show the regression analysis of the neural network model with desirable outcomes. The values of R^2^ are close to 0.9, indicating that the neural network model is strongly linear with the target values. This model has a great potential to be used in the palm oil industry for prediction purposes that can significantly improve the processes.

**Table 3:** Parameters of neural network model

Setting	Value
Network type	Feed-forward
Neurons in input layer	2
Number of hidden layers	2
Neurons in hidden layer	20
Neurons in output layer	1
Transfer function	Tangent sigmoid
Training function	Levenberg-Marquardt
Performance function	MSE

MSE=mean squared error

**Fig. 2:**
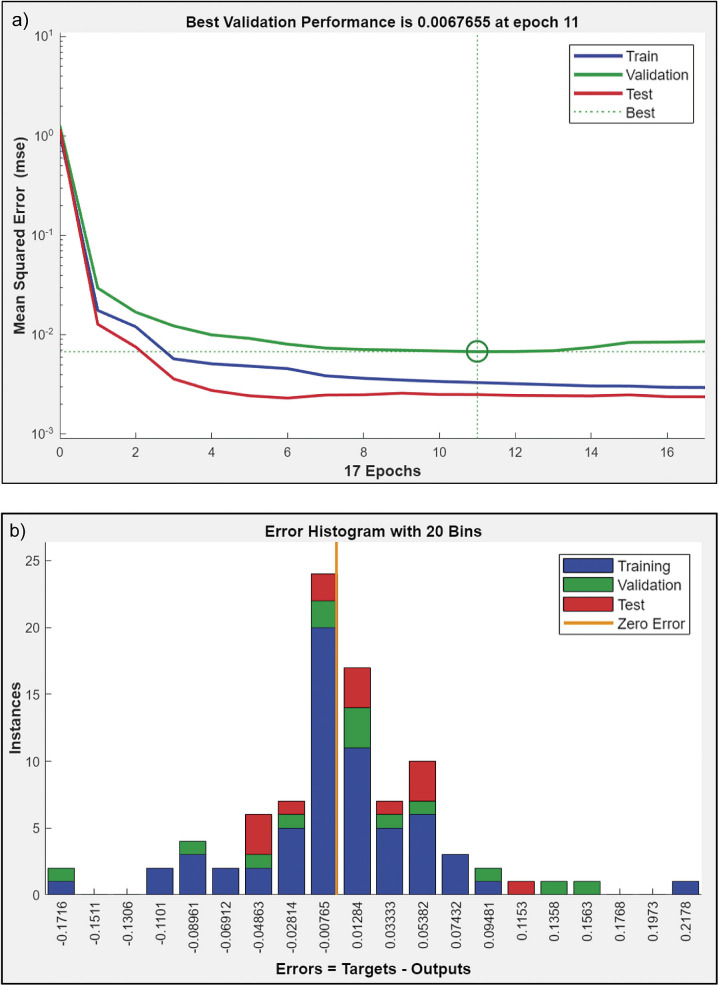
Artificial neural network (ANN) model: a) performance in terms of mean squared error (MSE), b) error histogram with 20 bins

The graph in Fig. 2a shows the MSE for training, validation and testing of the generated ANN model as a function of the epoch. An epoch in machine learning can be defined as the total number of iterations of all training data used in one cycle by the algorithm to train the model. The number of epochs is a crucial hyperparameter for the machine learning algorithm. Typical values for the number of epochs range from 10 to 1000 and can be increased further until the model error is sufficiently minimised (*37*). After running the neural network programme several times, the results with the best R^2^ were used. According to the results of the ANN model, the best validation performance was 0.0067655, which was observed at epoch 11. This means that during training, the neural network reached its optimal performance in the validation dataset at the 11^th^ iteration of training. After epoch 11, the validation of the data by the model cannot improve further, resulting in overfitting. The ideal ANN model is then developed by focusing on the MSE performance function and the coefficient of determination (R^2^). The MSE serves as an estimate of the average squared difference between the predicted and actual output values. A lower MSE value means smaller errors in predicting the actual outcomes of the dataset.

Fig. 2b shows an error histogram with 20 bins from the ANN model, which illustrates the quantitative differences between the target values and the output values after the development of the ANN model. In an error histogram, bins are used to group these differences (errors) into specific ranges, represented as vertical bars. Each bin shows how many predictions fall into a particular error range, which provides a clearer view of the overall accuracy of the model. In this histogram, the model has a notable bin with an error value of -0.00765, which contains around 20 data points where predictions fall within this range. This error value is close to zero, indicating that the model makes predictions with a small margin of error, which suggests a relatively high accuracy. Additionally, the zero-error line on the x-axis represents a perfect match between the predicted and target values, and illustrates the distribution of errors around this ideal point.

Overall, the results of this study highlight the crucial importance of integrating both ANN and SuperPro (*18*) simulation for effective management of a biogas plant due to their complementary strengths. ANN plays a vital role in accurately estimating specific parameters, such as H_2_S removal efficiency, using historical data and complex algorithms based on only two input variables: inlet temperature and biogas flow rate. In terms of performance, the ANN model in this study showed high predictive accuracy, which is confirmed by comparison with the work of Tan *et al.* (*33*), who reported an R^2^ value of nearly 1 and an MSE of 0.0002 for the prediction of H_2_S concentration. This capability is particularly valuable for optimising process performance and ensuring compliance with gas engine specifications, such as the H_2_S concentration in biogas for combustion.

On the other hand, SuperPro (*18*) simulation extends the predictive power by estimating parameters that are not monitored daily in the plant, such as COD, BOD, TSS, methane content, methane yield, *etc*. and other operational parameters including hydraulic retention time (HRT), organic loading rate (OLR) and recirculation flow rate. This broader scope enables a comprehensive understanding of overall process efficiency, environmental impact and operational optimisation. By integrating both ANN and SuperPro (*18*), these tools provide a robust framework for improving biogas plant management, enabling biogas plant engineers to anticipate variations in performance, address potential bottlenecks and take proactive measures to ensure stable and efficient bioscrubber operation. This integrated approach not only improves operational decision-making, but also contributes to the long-term economic viability of the palm oil industry. This aspect is further analysed in the next section.

### Techno-economic analysis

The techno-economic analysis (TEA) is a method for evaluating the economic potential of processes in industry (*36*). The economic analysis for this POME treatment plant was carried out using the economic evaluation function of the SuperPro Designer v. 9.0 software (*18*). The revenue of the processing plant comes primarily from the sale of the treated biogas, dried sludge and digested sludge to a composting plant. The reliability of this economic evaluation is up to date, as this report uses prices from 2022. Table 4 shows a brief report of the economic evaluation made with the simulation model. According to the sizing calculations performed with the software, the bioscrubber has a volume of 99.39 m^3^. The unit cost for a plant of this size is estimated at $250 000. The payback period is 7 years, which is consistent with a report by Loh *et al.* (*38*). This shows that the bioscrubber is economically feasible and offers a reasonable return on investment.

**Table 4:** Economic evaluation of the palm oil mill effluent (POME) treatment plant with bioscrubber for the removal of H_2_S

Parameter	Value
Total capital investment	$9,897,000
Capital investment charged to this project	$9,897,000
Operating cost	$2,849,000/year
Total revenue	$3,734,000/year
Gross margin	23.69 %
Return on investment	14.29 %
Payback time	7.00 years
IRR (after taxes)	7.27 %
NPV (at 7.0 % interest)	$131,000

IRR=internal rate of return, NPV=net present value

SUPPLEMENTARY METERIAL

In contrast, Tan *et al.* (*33*) reported a shorter payback period of 5.34 years for a local palm oil mill in Pahang, Malaysia. This shorter payback period was achieved through an optimisation study that emphasised the critical role of optimisation in increasing economic viability. This comparison highlights the potential to improve the effectiveness of the bioscrubber, particularly in increasing the efficiency of H_2_S removal. Additionally, optimising the conditions of the anaerobic digester can lead to the production of biogas with lower H_2_S concentrations. By focusing on parameters such as feedstock composition and operational conditions, future research can improve both H_2_S management and overall biogas quality. As a result, optimisation studies are essential for identifying and implementing strategies that minimise costs and maximise biogas production, which will ultimately contribute to more sustainable practices in the palm oil industry.

Furthermore, excessive H_2_S can shorten equipment lifespan and significantly increase maintenance costs (*35*). The use of an optimised bioscrubber with both ANN and SuperPro (*18*) will mitigate these effects by lowering H_2_S concentrations before the biogas enters critical equipment. This approach would reduce repair costs and extend the lifespan of the infrastructure of a biogas plant, promoting more efficient and cost-effective operations. By improving H_2_S management and optimising biogas quality, the integrated strategy ensures that the economic benefits of biogas production are fully realised.

### Limitations and future works

Although this study provides valuable insights into the integration of machine learning and SuperPro (*18*) simulations for biogas purification, it is important to recognise its limitations. A key limitation is the reliance on a dataset of 90 records, collected from a single palm oil mill, which may not capture the full range of variability in POME characteristics across different facilities. Although the dataset was collected under different operating conditions, its relatively small size may limit the generalisability of the model. To mitigate this, future studies could incorporate techniques such as k-fold cross-validation, which would help validate the reliability of the model across different data subsets and reduce overfitting.

The current simulation was limited to certain controllable parameters (*e.g.* inlet temperature, biogas flow rate), assuming that other operational aspects, such as the biofilm stability or packing media configuration, remain constant. This exclusion of certain biophysical interactions and operational variations may underrepresent how complex, interdependent factors influence bioscrubber efficiency. In future work, including a broader range of parameters or conducting a sensitivity analysis could help identify the primary variables that affect H_2_S removal efficiency to better match predictions under real-world conditions.

The performance of both the machine learning model and the SuperPro (*18*) simulation may also be affected by data quality, plant-specific operational conditions and the limited number of parameters considered in this analysis. Future research should consider extending the dataset to include data from multiple palm oil mills to improve the robustness and the potential to generalise the results. Additionally, conducting a feature importance analysis would help clarify the influence of individual parameters on model predictions, providing a clearer understanding of factors affecting the outputs of an integrated system.

To further improve prediction accuracy, future research could investigate the application of other machine learning models, such as support vector machines or deep learning techniques. Long-term performance evaluation under different seasonal or operational conditions would also be beneficial to evaluate the viability of the integrated approach in real-world settings.

## CONCLUSIONS

Simulation and prediction of the bioscrubber performance for biogas treatment are crucial for meeting the quality standards required for internal combustion engines used to generate electricity. This study demonstrates a novel integration of machine learning and process simulation in biogas plants based on palm oil mill effluent (POME). It provides a data-driven approach to optimise biogas purification and improve operational efficiency. Given the non-linear nature of many output responses in these processes, the selection of an appropriate machine learning algorithm to model the prediction of a unit is crucial. Therefore, an artificial neural network (ANN) was used to investigate the performance of the bioscrubber in the removal of H_2_S from biogas. Statistical parameters, including the coefficient of determination (R^2^) and mean squared error (MSE), were used to evaluate the accuracy of the ANN model. The model achieved an R^2^ of 0.90 and an MSE of 0.0068, confirming its high predictive accuracy and reliability without the need for further correlations or experimental measures. The POME treatment and biogas purification with the bioscrubber were simulated with SuperPro. Several parameters were observed and compared with industry data, with deviations of less than 10 %. These results demonstrate the high accuracy and prospects of using machine learning tools coupled with SuperPro simulation to predict the performance of bioscrubbers in the palm oil industry. The technoeconomic analysis also confirmed the economic feasibility of the bioscrubber, which offers a reasonable return on investment with a 7-year payback period. These results provide a scalable framework for improving biogas treatment processes, reducing reliance on trial-and-error approaches and supporting the wider adoption of AI-driven decision-making in industrial wastewater treatment. This integrated approach also enhances operational decision-making and supports the long-term economic sustainability of the palm oil sector. Future research should explore the application of other machine learning models, such as support vector machines or deep learning techniques, to further improve prediction accuracy.
